# Regulation of the *Yersinia* type III secretion system: traffic control

**DOI:** 10.3389/fcimb.2013.00004

**Published:** 2013-02-06

**Authors:** Rebecca S. Dewoody, Peter M. Merritt, Melanie M. Marketon

**Affiliations:** Department of Biology, Indiana UniversityBloomington, IN, USA

**Keywords:** type III secretion, injectisome, substrate specificity, YopK, YopE, YopN

## Abstract

*Yersinia* species, as well as many other Gram-negative pathogens, use a type III secretion system (T3SS) to translocate effector proteins from the bacterial cytoplasm to the host cytosol. This T3SS resembles a molecular syringe, with a needle-like shaft connected to a basal body structure, which spans the inner and outer bacterial membranes. The basal body of the injectisome shares a high degree of homology with the bacterial flagellum. Extending from the T3SS basal body is the needle, which is a polymer of a single protein, YscF. The distal end of the needle serves as a platform for the assembly of a tip complex composed of LcrV. Though never directly observed, prevailing models assume that LcrV assists in the insertion of the pore-forming proteins YopB and YopD into the host cell membrane. This completes a bridge between the bacterium and host cell to provide a continuous channel through which effectors are delivered. Significant effort has gone into understanding how the T3SS is assembled, how its substrates are recognized and how substrate delivery is controlled. Arguably the latter topic is the least understood; however, recent advances have provided new insight, and therefore, this review will focus primarily on summarizing the current state of knowledge regarding the control of substrate delivery by the T3SS. Specifically, we will discuss the roles of YopK, as well as YopN and YopE, which have long been linked to regulation of translocation. We also propose models whereby the YopK regulator communicates with the basal body of the T3SS to control translocation.

## Introduction

Pathogenic *Yersinia* species cause human diseases ranging from relatively mild intestinal disease for *Yersinia pseudotuberculosis* and *Yersinia enterocolitica* (Galindo et al., 2011) to bubonic plague for *Yersinia pestis* (Perry and Fetherston, 1997). Despite the differences in disease, virulence of these *Yersinia* species requires a conserved type III secretion system (T3SS) that has become a well-established model system for this form of protein secretion. Though first described in *Yersinia*, type III secretion is a conserved virulence factor amongst many human pathogens such as enteropathogenic *Escherichia coli* (EPEC), enterohemorrhagic *Escherichia coli* (EHEC), *Salmonella sp*., *Pseudomonas aeruginosa*, *Shigella flexneri*, and *Chlamydia sp.*, which collectively cause significant healthcare costs annually (for recent reviews of these organisms, see Schroeder and Hilbi, 2008; Agbor and McCormick, 2011; Dean, 2011; Wong et al., 2011). The T3SS has been described as a molecular syringe that delivers cytotoxic effectors into host cells. Because this virulence mechanism is conserved in so many pathogenic organisms, it makes an attractive target for new therapeutics. Interfering with effective delivery of effectors could have substantial consequences on disease pathology and, therefore, it is important to understand how bacteria sense cell contact in order to activate the T3SS and how both the fidelity and kinetics of effector delivery is coordinated.

In the *Yersinia*, genes of the T3SS are located on a 70 kb virulence plasmid, and the expression of these genes *in vitro* is controlled primarily by temperature and calcium concentration, a phenomenon referred to as the low calcium response (LCR) (Sample et al., 1987; Mehigh et al., 1989; Michiels et al., 1990; Straley et al., 1993). At ambient temperature, T3SS genes are not expressed. However, upon transfer of *Yersinia* cultures from 26°C to 37°C in the presence of millimolar calcium, conditions representing the mammalian host, T3SS genes are expressed at low levels and the injectisome is built (Straley et al., 1993). Chelating calcium from the medium *in vitro* causes the bacteria to undergo growth cessation and triggers massive up-regulation of T3SS gene expression along with secretion of T3SS substrates, known as Yops (*Yersinia* outer proteins) (Brubaker and Surgalla, 1964; Straley and Bowmer, 1986; Michiels et al., 1990; Straley et al., 1993; Petterson et al., 1996). *In vivo*, cell contact triggers polarized translocation of effector Yops into host cells (Rosqvist et al., 1994; Persson et al., 1995; Petterson et al., 1996; Lee et al., 1998) and growth cessation is thought to be overcome by additional environmental signals (Fowler and Brubaker, 1994; Fowler et al., 2009).

This review will focus on several factors that regulate the T3SS from both the proximal end of the injectisome inside bacteria and the distal end inside host cells. The *Yersinia* T3SS is a well-characterized archetype for this method of protein translocation, therefore, the data presented here will be compiled primarily from the *Yersinia* species: *Y. pestis* and the two closely related enteric pathogens *Y. pseudotuberculosis* and *Y. enterocolitica*. We will briefly review the building of the injectisome before discussing in more detail the regulation of translocation. We focus on the roles of YopN and YopK, which have been shown to play roles in governing substrate specificity during translocation. In addition, YopK, YopE, and YopT have been shown to play a role in T3SS regulation after they are injected into host cells, and those activities will be discussed as well.

## The making of an injectisome

**Figure 1:**
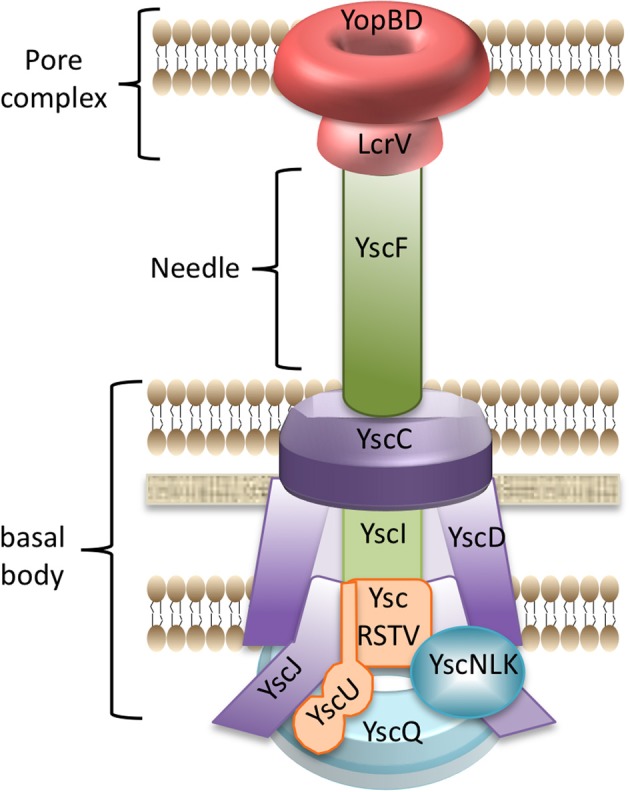
**Model of the injectisome.** Shown is a cartoon depicting the structural components of the *Yersinia* injectisome. Purple, scaffold proteins: YscC, YscD, YscJ; Orange, export apparatus proteins: YscR, YscS, YscT, YscU, YscV; Blue, cytoplasmic components: YscQ (C-ring) and YscN, YscL, YscK (ATPase complex); Green, YscI (rod) and YscF (needle); Red, pore complex: LcrV (needle tip complex) and YopB/YopD (translocation pore).

The “T3SS” or “injectisome” is homologous to the bacterial flagellum and is composed of several components that must be defined for the context of this review. YscC forms a ring in the bacterial outer membrane (the **OM ring**), and YscD and YscJ form a ring in the inner membrane (the **MS ring**). Together these proteins create a scaffold anchored within the peptidoglycan, and therefore, they will be referred to as **scaffold** proteins (Figure 1, purple). The **basal body** is the portion of the injectisome that spans the inner and outer membrane, including the scaffold proteins (YscCDJ) as well as proteins embedded within or connected to the scaffold: **export apparatus** (YscRSTUV Figure 1, orange), **ATPase complex** (YscNKL Figure 1, blue), and **C ring** (YscQ Figure 1, blue). The **needle** is the attached polymer of YscF that extends from the basal body into the extracellular milieu (Figure 1, green). Connecting the needle tip to the target host cell is a hypothetical structure called the **pore complex** (Figure 1, red), which is composed of LcrV at the needle tip and YopB/YopD forming a pore in the host cell membrane. The **injectisome** is the completed conduit comprised of the basal body, needle, and pore complex that allows translocation of Yops into host cells. Though a complete injectisome docked onto a host cell has never been observed, Figure 1 shows a model for how these components may be assembled.

### The basal body

The basal body formation begins with oligomerization of YscC, which forms the OM ring that spans the outer membrane and extends into the periplasm (Koster et al., 1997; Diepold et al., 2010). This is in contrast with the basal body of the flagellum which begins its assembly in the inner membrane and builds outward (Erhardt et al., 2010). After the OM ring is formed, a ring of YscD is assembled in the inner membrane and is thought to connect the outer and inner membrane rings (Spreter et al., 2009; Diepold et al., 2010; Ross and Plano, 2011). YscD then recruits YscJ, which oligomerizes to complete the MS ring (Yip et al., 2005; Hodgkinson et al., 2009; Diepold et al., 2010). With the assembly of these structures, a basic channel through the bacterial envelope is formed, which serves as a base for assembly of the remaining injectisome components.

An ATPase complex composed of YscN, YscK, and YscL forms on the cytosolic face of the basal body. YscN is the ATPase necessary for the secretion of substrates by the T3SS. YscL is a negative regulator of ATPase activity, while the function of YscK is as yet unknown (Blaylock et al., 2006). It has, however, been suggested that YscK may bridge the ATPase complex to the C ring. YscQ is assumed to comprise the C ring in *Yersinia* injectisomes based on homology to flagellar components (Driks and DeRosier, 1990; Khan et al., 1992; Kubori et al., 1997; Young et al., 2003; Thomas et al., 2006), co-localization with YscC in the membrane (Diepold et al., 2010) and association with the ATPase complex (Jackson and Plano, 2000). The ATPase complex and C ring associate with the scaffold proteins forming a nearly complete basal body (Diepold et al., 2010).

In a separate pathway, the export apparatus, composed of integral membrane proteins YscRSTUV (Allaoui et al., 1994; Fields et al., 1994; Minamino et al., 1994; Minamino and Macnab, 2000; Creasey et al., 2003; Melen et al., 2003; Ghosh, 2004; Spreter et al., 2009; Berger et al., 2010), assembles within the inner membrane independently of the scaffold proteins (Diepold et al., 2011). YscRST are necessary to promote the oligomerization of YscV. At this point, the assembly pathways converge and the export apparatus is recruited to YscJ in the MS ring of the scaffold (Diepold et al., 2011). With the joining of the scaffold, ATPase complex and export apparatus, the basal body is complete and is now capabable of exporting secretion substrates.

### The needle and the “early” stage

**Figure 2:**
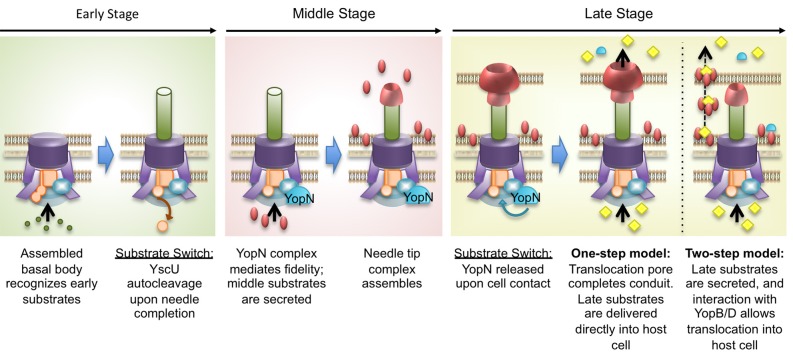
**Progression of injectisome assembly and activation.** In the **early** stage, the basal body recognizes early substrates (green) for secretion. These substrates are required for needle formation. Upon completing needle assembly, YscU (orange) undergoes autocleavage, which triggers a substrate specificity switch and transition to the **middle** stage. During this phase, YopN associates with the basal body to allow middle substrates (red) to be secreted, while rejecting late substrates. The middle substrates are required to form the tip complex and translocation pores. Upon cell contact, YopN is released from the basal body and secreted, triggering transition to the **late** stage. Two models are presented to depict the late stage. In the **One-step model**, the pore complex assembles at the tip of the needle to create a continuous channel, through which late substrates (yellow) are injected. In the **Two-step model**, late substrates are secreted into the extracellular space and then interact with pore proteins. The late substrate-pore complexes diffuse across the space and interact with the host membrane to deliver the late substrates.

Upon completion of the basal body, proteins necessary for needle assembly can be exported. We refer to this point as the “**early**” stage, because only “early” substrates are translocated (Figure 2). These include the first proteins to be secreted: YscIFPXO and YopR (Payne and Straley, 1998, 1999; Day and Plano, 2000; Agrain et al., 2005b; Blaylock et al., 2010). YscI is suggested to form a rod that allows substrate passage across the inner membrane (Allaoui et al., 1995; Sukhan et al., 2003; Marlovits et al., 2004; Wood et al., 2008; Sal-Man et al., 2012). The needle protein YscF is then secreted through the YscI rod (Diepold et al., 2012). Polymerization of YscF polymerizes forms the needle, which extends ~41 nm from the bacterium in *Y. pestis* or ~58 nm in *Y. enterocolitica*. The needle has an outer diameter of ~6–7 nm and an inner diameter of ~2–3 nm (Kubori et al., 1998; Blocker et al., 2001; Hoiczyk and Blobel, 2001; Journet et al., 2003).

Another T3S substrate, YopR, is important in the early stages of needle assembly (Allaoui et al., 1995; Lee and Schneewind, 1999), though its precise role is unclear. A *yopR* mutant secretes YscF, but cannot form a polymerized YscF needle (Riordan et al., 2008; Blaylock et al., 2010). In addition, YopR was found to interact with the ATPase protein YscN (Sorg et al., 2006) suggesting that YopR might regulate either secretion or polymerization of YscF.

YscP participates in needle assembly by regulating needle length (Payne and Straley, 1999; Stainier et al., 2000; Journet et al., 2003; Agrain et al., 2005a; Mota et al., 2005) and is, therefore, often referred to as the “ruler” protein. In support of its role in regulating needle assembly, a Δ*yscP* mutant over secretes YscF and forms needles of indiscriminate length, while secretion of “middle” or “late” substrates (pore complex components and Yop effectors) is severely compromised (Edqvist et al., 2003; Journet et al., 2003; Agrain et al., 2005a,b; Diepold et al., 2012).

YscX, YscY, and YscO are also required for export of early substrates, but their precise roles are unknown. Furthermore, though YscX and YscO are required for secretion of the early substrates, they do not appear to be secreted from the bacterium until needle assembly is complete (Payne and Straley, 1999; Day and Plano, 2000; Diepold et al., 2012). Some work has implicated YscO as a chaperone escort (Evans et al., 2006; Evans and Hughes, 2009; Ibuki et al., 2011), while data from Schneewind and colleagues suggest that YscO facilitates YscP interaction with the basal body during needle assembly (Riordan and Schneewind, 2008; Riordan et al., 2008). Therefore, YscO might assist YscP in regulating needle assembly, and afterward is exported. Both YscX and YscY associate with the export apparatus via YscV (Diepold et al., 2012), and YscY is thought to be the chaperone required for YscX secretion (Iriarte and Cornelis, 1999; Day and Plano, 2000). YscY has also been shown to bind to SycD, which is a chaperone facilitating secretion of the middle substrates YopB and YopD. The YscY-SycD interaction suggests a possible role for YscY in regulating secretion specificity (Broms et al., 2005). It is possible that once the needle is assembled, YscY releases YscX for secretion, and then YscY assists in secretion of the middle substrates that are required for the next stage of injectisome assembly.

In addition to its role in regulating needle length, YscP also interacts with YscU in the export apparatus to mediate a substrate specificity switch (Figure 2). Once the needle has reached the appropriate length, the switch is triggered, allowing recognition of middle and late T3SS substrates (YopBD and effector Yops, respectively). YscU has both a membrane spanning domain that anchors it in the inner membrane and a large cytosolic domain that is essential for substrate specificity (Allaoui et al., 1994; Edqvist et al., 2003; Sorg et al., 2007). It is thought that a conserved substrate specificity switch domain at the C-terminus of YscP interacts with the cytosolic domain of YscU to trigger autocleavage of YscU (Lavander et al., 2003; Agrain et al., 2005b; Sorg et al., 2007). This autocleavage is an essential step in the progression from the “early” to the “middle” stage. *yscU* mutants that cannot undergo proteolysis have severe consequences on expression and secretion of middle and late Yops and are also defective in preventing Yop secretion under restrictive conditions (+ calcium) (Riordan and Schneewind, 2008; Bjornfot et al., 2009).

### The calcium blockade

After the needle is built, and YscU undergoes proteolytic cleavage, the T3SS is capable of secreting middle and late substrates: middle substrates are those comprising the pore complex, while late substrates are the effectors that are delivered into host cells. However, in medium containing calcium (or in the absence of cell contact), secretion of late substrates is largely prevented due to the action of the YopN-TyeA-YscB-SycN complex, also referred to as the calcium plug (Yother and Goguen, 1985; Forsberg et al., 1991). During growth of wild type *Yersinia* in calcium-replete medium, secretion of early and middle T3SS substrates into the extracellular milieu is readily observed, whereas the late Yops are not released in large amounts until either calcium chelation or contact with a host cell occurs (Lee et al., 1998). Therefore, we use “**middle**” stage to refer to the T3SS after the completion of needle assembly and the YscP-YscU substrate switch has been triggered, but before contact with a host cell has been made (Figure 2).

Support for the “calcium block” model stems from observations that deletion of any of the YopN-TyeA-YscB-SycN complex genes results in the massive secretion of both middle and late Yops in both the presence and absence of calcium *in vitro*, a defect known as a calcium blind phenotype (Forsberg et al., 1991; Skrzypek and Straley, 1995; Day and Plano, 1998; Iriarte et al., 1998; Jackson et al., 1998; Cheng and Schneewind, 2000; Cheng et al., 2001; Sundberg and Forsberg, 2003). During infection of tissue culture cells, this defect manifests as a loss of specificity phenotype whereby Yops are secreted into the medium as well as translocated into host cells (Boland et al., 1996; Lee et al., 1998; Cheng and Schneewind, 2000; Cheng et al., 2001; Day et al., 2003). TyeA binds to the C-terminal half of YopN and acts as a negative regulator of YopN secretion, helping to maintain a secretion incompetent state in the presence of calcium (Iriarte et al., 1998; Cheng et al., 2001; Ferracci et al., 2004, 2005; Schubot et al., 2005). YscB and SycN bind to each other in the bacterial cytosol and then to an N-terminal region of YopN (Day and Plano, 1998; Cheng et al., 2001). To release the YopN-TyeA block once cell contact is made, YscB and SycN act as chaperones to mediate YopN export through the injectisome.

### The “middle” stage and translocon assembly

The “**middle**” stage is distinguished by the secretion of the translocators (or “middle” Yops), which include LcrV, YopB, and YopD. These proteins are secreted into the extracellular milieu during tissue culture infection and during *in vitro* growth in calcium-replete medium (Lee et al., 1998; Cheng and Schneewind, 2000; DeBord et al., 2001; Houppert et al., 2012), indicating that they are secreted prior to cell contact. LcrV is secreted and polymerizes at the distal end of the YscF needle forming a pentameric needle tip complex (Mueller et al., 2005) that is necessary for translocation of late substrates (Goure et al., 2004, 2005). The pore proteins, YopD and YopB, contain one and two transmembrane domains, respectively, and are capable of inserting themselves into the host cell membrane (Hakansson et al., 1993; Rosqvist et al., 1995; Hakansson et al., 1996; Neyt and Cornelis, 1999; Montagner et al., 2011). The LcrV tip complex is thought to act as a platform for insertion of YopB and YopD into the host cell membrane (Goure et al., 2004, 2005; Picking et al., 2005; Broz et al., 2007; Mueller et al., 2008), and it is through this pore complex that late Yops are thought to be delivered. The properties and proposed functions of the pore complex proteins have been recently reviewed (Mattei et al., 2011).

Though a complete conduit between the needle and the host cell has never been observed, there is evidence from *Yersinia* and *Shigella* studies to support the idea of a translocator complex that connects the needle with the host membrane. First, the YopB/YopD translocators (IpaB/IpaC in *Shigella*) can be isolated from host membranes, and LcrV (IpaD in *Shigella*) is required for insertion of YopB/YopD (IpaB/IpaC) (Goure et al., 2004, 2005; Picking et al., 2005; Broz et al., 2007). Second, IpaB has been observed on the tip of needles as part of the tip complex (Ide et al., 2001; Olive et al., 2007; Veenendaal et al., 2007). Most recently, YopD has been detected in purified needle preparations in an LcrV-dependent manner (Ligtenberg et al., 2012). Furthermore, there is a direct correlation between the length of the needle, the distance between the bacteria and host cell, and the ability to inject Yops: the effect of changing the length of the surface adhesin YadA on *Y. enterocolitica* can be counteracted by changing the length of the needle (Mota et al., 2005). In our schematic (Figure 2, One-step model), assembly of the translocation pore complex at the distal end of the needle would form a complete channel connecting the bacterium to the host cell, and this would trigger the “cell contact signal” that releases the YopN regulatory blockade. This signifies transition to the “**late**” stage, whereby the “late” Yops (effectors) are now delivered directly into host cells.

For many years, T3SS models have depicted pore complex formation as the final step in completing a channel between the bacterium and the host cell (Figure 2, One-step model). However, this hypothetical model has been recently challenged by the demonstration that extracellular late Yops, in association with YopB and YopD, could be translocated into host cells (Akopyan et al., 2011; Edgren et al., 2012) (Figure 2, two-step model). Akopyan and colleagues found that middle and late Yops were present on the surface of *Y. pseudotuberculosis* cells in calcium-replete medium, indicating that bacteria are coated with Yops prior to host cell contact (Akopyan et al., 2011; Edgren et al., 2012). This is in contrast to prior work showing that late Yops were not secreted into the medium prior to cell contact (Rosqvist et al., 1994; Sory and Cornelis, 1994; Persson et al., 1995; Lee et al., 1998; Cheng and Schneewind, 2000; DeBord et al., 2001). Although the observations do not rule out the one-step “conduit” model of translocation, they do suggest that late Yops can also be delivered into host cells by a two-step method with an extracellular intermediate step. It is possible that while middle Yops are secreted in readily detected amounts prior to cell contact, there is “leaky” low level secretion of late Yops, that are able to aggregate onto the bacterial surface and associate with YopB/D translocators. Upon cell contact (or calcium depletion) the YopN blockade is released and massive amounts of late Yops are then exported and are readily detected. Notably, the two models are not mutually exclusive, and in both models, YopB and YopD are required for late Yop delivery.

### Transition to the “late” stage

There is an abundance of data to support a model in which there is a conformational change in the needle that is triggered by external stimuli such as cell contact or calcium depletion. This change would be transduced to the basal body, which then triggers release of the TyeA/YopN complex and subsequent translocation of YopN, thereby relieving its blockade on late Yop export (Ferracci et al., 2004, 2005; Torruellas et al., 2005; Hamad and Nilles, 2007; Davis et al., 2010). In support of this model, a crystal structure of the YopN/TyeA complex revealed that TyeA has a conserved C-terminal helix that could potentially interact with components of the basal body (Schubot et al., 2005). Additionally, a YopN-TyeA hybrid fusion protein expressed in *Y. pestis* is completely functional for calcium sensing and Yop secretion regulation (Ferracci et al., 2004). This suggests that YopN binding of TyeA in itself does not block the injectisome; rather that TyeA tethering of YopN to the basal body causes the blockage. In further support of this, YopN mutants that constitutively block secretion require the presence of TyeA, but not chaperones YscB or SycN (Ferracci et al., 2005). This would be consistent with a conformation change in the T3SS that releases TyeA, thereby relieving the late Yop secretion block. Previous work investigating needle protein YscF has shown that the needle itself also can act as a calcium sensor, since mutations can be isolated that correlate with different stages of regulation (Torruellas et al., 2005; Davis et al., 2010). Therefore, contact with a host cell could trigger a cascade of conformational changes throughout the T3SS such that the information is transduced through the needle and down to the basal body. Upon receiving the signal, the TyeA tether is broken, which allows delivery of YopN into host cells to relieve the block on late Yop secretion.

## Regulators of injection: YopK, YopE, and YopT

### YopK: a regulator of translocation rate

YopK was first discovered in *Y. pestis* by Straley and Bowmer in a screen searching for LCR genes (Straley and Bowmer, 1986). It was later determined that YopK (named YopQ in *Y. enteroc*olitica) is present in all three pathogenic *Yersinia* spp. with a high degree of sequence homology (Fernandez-Lago et al., 1994; Holmstrom et al., 1995a). YopK is a T3SS substrate and has an N-terminal secretion signal within the first 10 residues (Michiels and Cornelis, 1991; Anderson and Schneewind, 1999), and expression of YopK is regulated by calcium and temperature as are other effector Yops (Straley and Bowmer, 1986; Holmstrom et al., 1995a). YopK was found to be important for the mouse model of *Y. pestis* infection as a Δ*yopK* mutant is severely attenuated compared to wild type (Straley and Bowmer, 1986; Straley and Cibull, 1989; Holmstrom et al., 1995a). The Δ*yopK* mutant poorly colonizes the liver and is quickly cleared from the spleen (Straley and Cibull, 1989). Likewise, the *Y. pseudotuberculosis* Δ *yopK* mutant colonizes Peyer's patches, but due to rapid clearance cannot colonize the spleen (Holmstrom et al., 1995a,b). YopK has no known enzymatic activity, and the *yopK* mutant retains the ability to cause cytotoxicity and prevent phagocytosis (Holmstrom et al., 1995a,b).

Understanding the role of YopK during infection was a challenge since it is expressed at low levels. Attempts to visualize YopK during infection by immunofluorescence only showed a YopK signal within bacteria near the zone of contact with the host cell (Holmstrom et al., 1995a,b, 1997). Differential detergent fractionation was also unable to accurately decipher YopK localization as it is found in the digitonin pellet fraction containing adherent bacteria, along with host cell membranes and organelles (Lee and Schneewind, 1999). With the creation of a GSK tag reporter system, Garcia et al. finally demonstrated that YopK from *Y. pestis* is injected into host cells during infection (Garcia et al., 2006). *Y. pseudotuberculosis* YopK translocation was also observed recently using a β-lactamase (Bla) reporter (Thorslund et al., 2011). Because YopK is expressed at low levels in comparison to other Yops (Holmstrom et al., 1995a) it may be that the amount of YopK delivered to host cells is below the limit of detection by previous methods.

Early work found that a *Y. pseudotuberculosis yopK* mutant infection induced cytotoxicity (seen as cell rounding) in host cells more rapidly than wild type, while overexpression of YopK resulted in a lack of cytotoxicity (Holmstrom et al., 1997). This phenotype led researchers to investigate the possibility that YopK is involved in the regulation of translocation. Immunofluorescence microscopy on infected culture cells revealed that a *Y. pseudotuberculosis yopK* mutant injects a larger quantity of YopE and YopH into host cells, whereas overexpression of YopK inhibits translocation of YopE and YopH (Holmstrom et al., 1997). This suggests that the hyper-cytotoxicity phenotype of a *yopK* mutant is due to an increased concentration of YopE, a GTPase activating protein (GAP), in host cells (Holmstrom et al., 1997; Aili et al., 2002). It was found that YopK has no role in transcription or expression of effectors YopE or YopH, supporting the hypothesis that YopK activity is restricted to regulating translocation (Holmstrom et al., 1997).

#### Indirect measurements of translocation regulation

Shortly before the characterization of YopK by Holmstrom and colleagues, a contact-hemolytic assay, previously utilized for pore-forming toxins (Sansonetti et al., 1986), was modified to assess the membrane disrupting ability of *Yersinia* translocator protein YopB (Hakansson et al., 1996). In this assay, red blood cells are infected with *Yersinia* strains and the subsequent release of hemoglobin provides a metric for pore formation. In addition, differentially sized sugars were incubated with infected RBCs to estimate the size of lytic membrane pores formed during infection (Bhakdi et al., 1986; Braun et al., 1987). It was shown using [C^14^] sucrose that an influx of sugar into host cells occurs upon effector-induced membrane disruption and that the influx can be blocked by incubation with sugar moieties larger than the membrane pore (Bhakdi et al., 1986; Braun et al., 1987). Because mutations in *yopK* (as well as *yopE* discussed below) gave rise to higher levels of effector translocation, it was hypothesized that the phenotype may be related to a change in translocation pore size. As a result, several studies were undertaken to investigate this possibility.

Using the contact-hemolysis assay, Holmstrom and colleagues found that a *Y. pseudotuberculosis* Δ*yopK* mutant showed a significant increase in hemoglobin release compared to wild type infection of erythrocytes and that hemolysis was dependent on the presence of YopB (Holmstrom et al., 1997). Complementation of YopK restored wild type levels of hemoglobin release, and overexpression of YopK also rescued the hemolytic phenotype of a multi-Yop mutant (Holmstrom et al., 1997). Incubation of infected erythrocytes with differentially sized sugars indicated a wild type pore size of 2.2 nm, an increased pore size of 3.5 nm in the absence of YopK, and a decreased pore size of >1.2 nm when YopK was overexpressed (Holmstrom et al., 1997). While these lytic assays do not directly measure translocation of Yops, they do suggest that YopK can control translocation pore size, which can in turn have an effect on translocation.

Lactate dehydrogenase (LDH) release is a common cytotoxicity assay that is often used to measure membrane disruption and/or cell death by detecting the concentration of cytosolic LDH that is released into the extracellular medium. Several groups have used this assay to test lytic pore formation as correlate of translocation regulation. In contrast to the contact-hemolysis assay, the Δ*yopK* mutant had no effect on LDH release from HeLa cells or bone marrow derived macrophages during *Y. pseudotuberculosis* infection (Aili et al., 2008). This discrepancy could be due to differences between nucleated and non-nucleated cells, or it could reflect differences in cytoskeletal networks and actin remodeling in various cell types.

It is not yet clear how the observed changes in “pore” size (as determined by lytic pore formation assays) correlate with changes in translocation levels. If the translocation pore is indeed larger, then is the internal diameter of the needle larger to accommodate increased flow of effectors? Considering prevailing models in which the translocation pore complex is connected to the basal body through the needle, a change in the pore should trigger compensatory changes throughout the injectisome. Another possibility is that the assays which measure pore formation do not directly measure the pores that are actively translocating, and instead, the results could simply reflect an altered conformation that is perhaps less stable or more flexible, and therefore appears larger. If the pore size is not physically larger, it may be that the altered conformation of the pore, and by extension the rest of the injectisome, triggers a change in the export apparatus and/or ATPase complex such that secretion substrates are recognized and initiated through the channel at a faster rate.

#### Direct measurement of translocation regulation

To better understand the mechanism by which YopK regulates translocation, a time course infection was performed using a Bla reporter system (Dewoody et al., 2011). This approach relies on detection of Bla reporter fused to an effector Yop, which is injected into host cells in a T3SS-dependent manner (Marketon et al., 2005; Dewoody et al., 2011). Using a fluorescent Bla substrate, translocation of the Yop-Bla reporter can be detected quantitatively by flow cytometry, thus providing a direct measure of translocation. When infections are synchronized, this approach also affords a comparison of translocation efficiency or rate by different strains. Measuring translocation rates with a YopM-Bla reporter revealed that wild type *Y. pestis* and a Δ*yopK* mutant initiate injection with similar efficiency (Dewoody et al., 2011). However, a difference in translocation between the two strains can be seen as soon as 1.5 h post-infection, showing that both strains inject the same number of cells, but the Δ*yopK* strain injects more YopM-Bla reporter per cell and does so at a faster rate (Dewoody et al., 2011).

Observing that YopK is translocated into host cells and also plays a prominent role in controlling the injection of other effectors prompted the question of whether YopK performs this regulatory function within the attached bacterium or within target cells. Injection of YopK into host cells is essential to its ability to regulate translocation of effector Yops because a non-injectable form of YopK (Gst-YopK) does not complement a Δ*yopK* mutant (Dewoody et al., 2011). In addition, YopK expressed solely in the host cell can restore regulation of injection during infection with a Δ*yopK* mutant (Dewoody et al., 2011). Taken together, these data suggest that YopK is injected into host cells, and then acts as a strong down-regulator of translocation by transmitting a signal back to the bacterium to slow translocation.

#### YopK protein-protein interactions

Given that YopK works within target cells to regulate translocation, along with the evidence suggesting that it affects pore size or conformation, researchers began investigating whether YopK interacts directly with the translocation pore complex. It was shown that YopK could be immunoprecipitated with YopB from cells infected with *Y. pseudotuberculosis*, providing the first evidence of a direct interaction between YopK and the pore (Brodsky et al., 2010). In another study, infection of erythrocytes with *Y. pseudotuberculosis* resulted in YopK, YopB, and YopD being pulled down from red blood cell membranes, suggesting that they form a complex in target cell membranes (Thorslund et al., 2011). In the same study, YopK interacted with His-tagged YopD from bacterial supernatants, but seemingly not YopB (Thorslund et al., 2011). Recent data regarding *Y. pestis* YopK is in agreement with the *Y. pseudotuberculosis* data (Dewoody et al., 2012). In this study, YopK was expressed from a eukaryotic expression vector within host cells and then infected with a *Y. pestis* Δ*yopK* mutant. The infected cells were then lysed, and YopK along with any interacting proteins were co-immunoprecipitated with affinity purified YopK antibody. YopK, which was only present within host cells, was able to pull down YopD but not YopB (Dewoody et al., 2012). Collectively, these data support a model for YopK interacting directly with YopD of the translocation pore within the cytosol of targeted cells thereby influencing the function of the injectisome.

#### YopK regulates translocation fidelity

Control of substrate fidelity from within the bacteria by YopN has been known for quite some time (Brubaker and Surgalla, 1964; Michiels et al., 1990; Rosqvist et al., 1994; Persson et al., 1995; Boland et al., 1996; Day and Plano, 1998; Cheng and Schneewind, 2000; Cheng et al., 2001). A Δ*yopN* mutant aberrantly secretes and injects both middle and late Yops during infection (Boland et al., 1996; Cheng and Schneewind, 2000). Recent work in our lab has revealed a similar but distinct phenotype for the *Y. pestis* Δ *yopK* mutant (Dewoody et al., 2012). These studies build upon an earlier observation that showed, using immunofluorescence, that the middle substrate, YopD, is injected into host cells by a *yopK* mutant (Francis and Wolf-Watz, 1998). By fractionating infected cells and immunoblotting for a series of Yops representing early, middle, and late T3SS substrates, we found that both middle (LcrV, YopD, and YopB) and late (YopE, YopH, YopM, and YopN) Yops are injected into host cells, while normal rejection of early substrates (YopR and YscF) is maintained. This observation was confirmed through the use of middle Yop reporters, which were created by fusing Bla to YopD and YopB. We found that YopD-Bla and YopB-Bla are injected by the *yopK* mutant, but not by wild type or the *yopE* mutant (Dewoody et al., 2012), which is in agreement to previous work (Francis and Wolf-Watz, 1998). Thus, it appears that like YopN, YopK is also required to maintain fidelity of translocation such that only late Yops are delivered into host cells.

**Figure 3:**
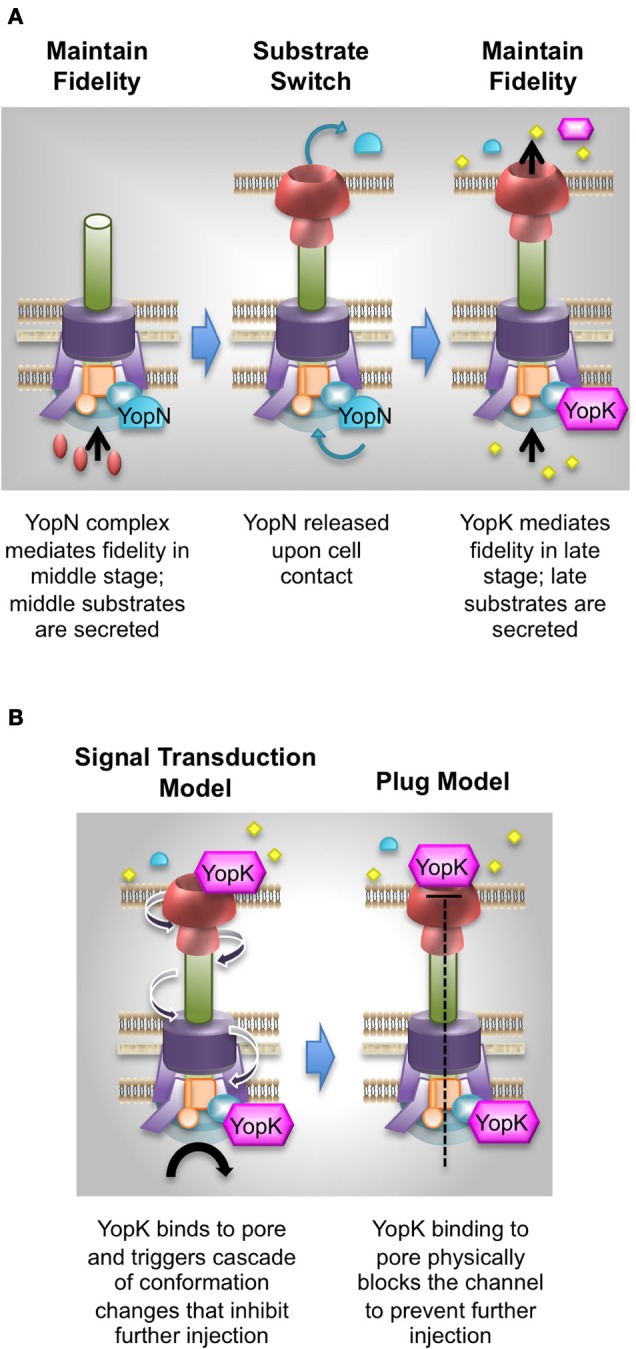
**Models for YopK functions. (A)** Controlling fidelity. During the middle stage, YopN is associated with the basal body to prevent premature release of late substrates. This blockade is released upon cell contact by translocating YopN into host cells. YopK now associates with the basal body to prevent aberrant injection of middle substrates. **(B)** An OFF switch. During the late stage, YopK is injected into the host cell and acts to down-regulate injection of the other late substrates. Two models are shown to depict how this may happen. In the **Signal transduction model**, YopK would interact with the pore complex and cause a conformational change in the pore, which then triggers structural changes along the length of the injectisome to provide a signal to the basal body. Further transport of late substrates is then inhibited. In the **Plug model**, such structural changes are not necessarily induced. Rather, YopK binding to the pore causes a physical blockade to the channel such that substrates cannot pass through the pore.

The phenotype of a *yopK* mutant injecting middle substrates into host cells is quite novel and indicates that YopK is a bifunctional protein capable of regulating fidelity as well as rate of Yop injection. Importantly these properties of YopK are distinct and genetically separable, since a point mutation (YopK_D46A_) abolishes the ability of YopK to control the rate of late Yop delivery but is still able to inhibit injection of middle Yops (Dewoody et al., 2012). Notably, though the YopK_D46A_ mutant is not able to regulate late Yop injection, it is still able to associate with YopD and therefore presumably maintains an interaction with the translocation pore (Thorslund et al., 2011; Dewoody et al., 2012). Furthermore, although expression of YopK within host cells can complement the *yopK* mutant *in trans*, by lowering the injection levels of late Yops, it cannot prevent injection of middle Yops. Together these data indicate that the two functions of YopK are genetically and spatially distinct. We currently favor a model in which YopK interacts with the basal body during the “late” stage in order to ensure that only late Yops travel through the injectisome (Figure 3A).

As YopK is itself injected at low levels into host cells, its appearance within the host cytosol and localization to the translocation pore could provide a negative feedback signal to down-regulate further effector delivery (Figure 3B). Why provide such a negative feedback signal? Perhaps this would provide an external cue to initiate detachment from the host cell, which could facilitate dissemination and subsequent re-colonization *in vivo*. Additionally, it would be prudent to prevent over-injection of cytotoxic effectors, since that would likely cause an early inflammatory response, which could be counterproductive to disease progression. In support of this, *yopK* mutants are known to have colonization and dissemination defects (Straley and Bowmer, 1986; Straley and Cibull, 1989; Holmstrom et al., 1995a,b). Furthermore, a *Y. pseudotuberculosis yopK* mutant triggers caspase-1 and inflammasome activation (Brodsky et al., 2010), while a *Y. pestis yopK* mutant causes increased apoptosis and death of alveolar macrophages (Peters and Anderson, 2012).

How might YopK down-regulate late Yop injection? We envision two basic options for YopK activity within host cells: (1) YopK binds to the translocation pore to trigger conformational changes that influence substrate recognition and/or transport by the basal body (Figure 3B, signal transduction model), (2) YopK binds to the translocation pore and acts as a plug to block the channel and prevent further late Yop translocation (Figure 3B, plug model). If YopK acts as a plug, then one would predict that mutations abolishing YopK function would also abolish YopK binding to the pore proteins. The combined observations that YopK can bind to the translocation pore component YopD, and that the YopK_D46A_ mutant loses the ability to control late Yop injection without losing its association with YopD, suggest that YopK binding to YopD might create a structural change in the pore complex that is transduced to the bacterial side of the injectisome. Much more work is necessary to understand how YopK performs its two regulatory functions, and in doing so, it may be possible to determine whether an analogous regulator exists in other organisms.

### YopE: the GTPase activating protein

YopE is one of the best characterized of the effector Yops, perhaps because it is one of the most highly translocated into host cells and is essential to virulence (Straley and Cibull, 1989; Lee et al., 1998). YopE is highly cytotoxic as seen by a characteristic cell rounding as soon as 15 minutes post-infection (Straley and Cibull, 1989; Rosqvist et al., 1991). Cell rounding is caused by disruption of the host cell cytoskeleton, in particular the actin microfilaments in stress fibers (Rosqvist et al., 1991). In addition to causing cell rounding, the actions of YopE on the cytoskeleton also serve to immobilize the cells and prevent phagocytosis. YopE acts as a GAP by maintaining specific GTPases in an inactive, or GTP-hydrolyzed, state using the conserved arginine finger motif associated with such domains (Black and Bliska, 2000; Von Pawel-Rammingen et al., 2000). Small Rho GTPases RhoA, Rac1, and Cdc42 are inactivated by YopE *in vitro*, although Cdc42 does not seem to be a target *in vivo* (Black and Bliska, 2000; Aili et al., 2006). During infection, the cytosolic pool of activated RhoG and Rac1 is quickly depleted when infected with *Y. pseudotuberculosis* expressing YopE (Wong and Isberg, 2005; Mohammadi and Isberg, 2009). In addition to its anti-phagocytosis role, YopE is also implicated in controlling pore formation and regulating translocation, and these are all thought to involve actin regulation. Each topic is addressed below.

#### Anti-phagocytosis

YopE plays several roles in the host cell in addition to (or due to) actin regulation. Because yersinae proliferate extracellularly, it is essential to block phagocytosis after contact with host cells. *Y. pseudotuberculosis* YopE was shown to play a role in preventing engulfment by macrophages (Rosqvist et al., 1990). To assess the role of GAP activity in phagocytosis, a mutation of the essential arginine residue (YopE_R144A_) was evaluated. Although YopE_R144A_ was translocated efficiently, it could not prevent phagocytosis by HeLa cells (Black and Bliska, 2000). This indicates that YopE's GAP activity is essential for its anti-phagocytic activity.

#### Inhibition of lytic pore formation

Another facet of YopE's function is the ability to inhibit the formation of lytic pores. Several groups have shown that infection with a *Y. pseudotuberculosis* Δ*yopE* mutant released more LDH from HeLa cells than did a wild type infection, and that the phenotype was dependent on the GAP domain of YopE (Viboud and Bliska, 2001; Aili et al., 2006, 2008; Viboud et al., 2006; Mejia et al., 2008). In contrast to LDH release assays, a contact-hemolysis assay showed *Y. pseudotuberculosis* Δ*yopE* caused hemoglobin release similar to wild type infection (Holmstrom et al., 1997). Interestingly, as noted above the same assays performed on *yopK* mutants also yielded contrasting results, but in the case of *yopK*, it was the hemolysis assay that showed a phenotype. The difference in phenotypes may be related to differences in the cytoskeleton of each cell type, as RBCs and epithelial cells have different actin cytoskeletal networks (Nans et al., 2011). These observations support the idea that YopE and YopK work by different mechanisms within host cells to control translocation pores.

To further analyze the role of YopE GAP activity in lytic pore formation, Viboud et al. transfected HeLa cells with constitutively active forms of RhoA or Rac1 that cannot hydrolyze bound GTP. When HeLa cells were transfected with activated GTPases before infection, ectopic expression of YopE could no longer complement the *yopE* mutant and resulted in high LDH release (Viboud and Bliska, 2001). Thus, constitutively active GTPases in the host cell blocked the ability of YopE to function. Additionally, actin regulation was found to be important for the lytic pore inhibition, since the presence of actin polymerization inhibitors cytochalasin D and latrunculin B blocked LDH release during infection with the Δ *yopE* mutant (Viboud and Bliska, 2001). Similar results were found with *Clostridium difficile* ToxB, an inhibitor of Rho, Rac, and Cdc42 GTPases (Mejia et al., 2008). Taken together, these data suggest that deactivation of small GTPases and disruption of host cell actin are essential to the mechanism by which YopE blocks lytic pore formation in host cell membranes.

#### Translocation regulation

Δ*yopE* mutant strains have been shown to over-inject Yops into host cells during infection. Fractionation of infected cells has shown that a *Y. pseudotuberculosis* Δ*yopE* mutant injects higher levels of YopH, and the corresponding YopE_R144A_ GAP mutant over-injects both YopH and YopE, (Aili et al., 2006, 2008; Isaksson et al., 2009). In *Y. pestis*, YopE was shown to regulate translocation using the YopM-Bla reporter (Dewoody et al., 2011). A Δ*yopE* mutant showed an approximately 50% increase in YopM-Bla injection compared to infection with wild type. This phenotype is dependent on YopE's GAP ability as ectopic expression of YopE_R144A_ could not complement the Δ*yopE* parent strain (Dewoody et al., 2011).

In summary, YopE plays a role in three major aspects of *Yersinia* infection: anti-phagocytosis, repression of lytic pore formation, and translocation regulation. Each of these processes depends on small Rho GTPase deactivation and thus inhibition of actin polymerization. What has yet to be determined is whether one of these roles is the major function of YopE or if each is essential for infection. Furthermore, since there is no evidence for YopE binding directly to the translocation pore complex, any affect it has on the injectisome pore and translocation must be indirect. Finally, direct evidence for connections between YopE, the actin cytoskeleton, and changes to the injectisome have yet to be revealed.

### YopT: the cysteine protease

YopT was first discovered in *Y. enterocolitica* as an effector that was translocated into host cells where it disrupted actin stress fiber and caused cytotoxicity (Iriarte and Cornelis, 1998). It is expressed in most *Y. enterocolitica* and *Y. pestis* strains, but only a portion of *Y. pseudotuberculosis* strains (Viboud and Bliska, 2001; Aepfelbacher, 2004; Viboud et al., 2006). YopT is dispensable for infection of Peyer's patches by *Y. enterocolitica*, and a Δ*yopT* mutant shows a slight increase in virulence (Iriarte and Cornelis, 1998; Trulzsch et al., 2004). YopT is not necessary for *Y. pseudotuberculosis* infection, but can partially restore the virulence of a Δ*yopE* mutant, suggesting some degree of redundancy between the two cytotoxins (Viboud et al., 2006). This redundancy may reflect the fact that both proteins target small Rho GTPases for inactivation, albeit through different methods. Though no direct role for YopT in translocation has been shown, its activity on the host cytoskeleton suggests that, like YopE, it contributes to the regulation of Yop injection.

#### Anti-phagocytosis and lytic pore inhibition

YopT is a cysteine protease and shares the conserved invariant C/H/D residues necessary for proteolytic function (Shao et al., 2002). During infection, YopT cleaves the prenyl modifications of membrane-bound RhoA, RhoG, Rac, and Cdc42. Proteolysis releases the GTPases from the membrane thereby disrupting actin structures such as stress fibers and phagocytic cups (Iriarte and Cornelis, 1998; Zumbihl et al, 1999; Grosdent et al., 2002; Aepfelbacher et al., 2003). Infections with *Y. enterocolitica* strains expressing YopT have shown that activated RhoA is released from the host membrane (Zumbihl et al, 1999; Sorg et al., 2001). Therefore, like YopE, YopT functions to disrupt actin regulation. In addition, YopT creates a pool of activated GTPases located in the nucleus (Shao et al., 2002, 2003; Aepfelbacher et al., 2003; Wong and Isberg, 2005; Mohammadi and Isberg, 2009). The significance of this during infection is unknown; however, it cannot be essential, as *yopT* mutants do not have a virulence defect *in vivo* (Iriarte and Cornelis, 1998; Trulzsch et al., 2004; Viboud et al., 2006).

Due to its ability to regulate host actin regulation, it is not surprising that YopT has a role in preventing phagocytosis and lytic pore formation. Expression of *Y. pseudotuberculosis* YopT alone is able to significantly reduce LDH release and lytic pore formation of infected HeLa cells (Viboud et al., 2006). YopT is also able to rescue the LDH release phenotype of a Δ*yopE* mutant, but not when activated Rho or Rac are expressed in host cells (Viboud and Bliska, 2001). This indicates that while YopT and YopE function by distinct mechanisms, there is overlap of the resultant actin regulation phenotypes.

Inhibition of phagocytosis by YopT was measured using both a gentamicin protection assay and double immunofluorescence staining. Expression of YopT in a Δ*yopEHJT Y. pseudotuberculosis* background resulted in a small decrease in phagocytosis when expressed at native levels and larger inhibition when overexpressed (Viboud et al., 2006). In either case, the anti-phagocytic effect of YopT was not as potent as that of YopE (Viboud et al., 2006). Interestingly, infection of macrophage lines with a *Y. enterocolitica* Δ*yopT* mutant resulted in phagocytosis well above that of wild type and not significantly different from a Δ*yopE* mutant (Grosdent et al., 2002). In fact, when bacteria were opsonized before infection, the Δ*yopE* mutant had no affect on phagocytosis while the Δ*yopT* mutant showed significantly more internalization (Grosdent et al., 2002). The reasons for the differences in observed phenotypes are not entirely clear; however, as with observations regarding pore formation with *yopK* and *yopE* mutants, the host background plays a confounding role in the functions of these proteins. Given that different eukaryotic cell types lead to different phenotypes for these mutants, future work may be able to utilize this knowledge to gain insight into the underlying mechanisms.

## Perspectives

### Translocation regulation

Translocation of effectors by the T3SS is tightly regulated to optimize the infectious process of disease. It is well-established that YopN functions prior to cell contact in order to prevent the premature release of late Yops into the medium, thereby providing a measure of fidelity to substrate recognition by the T3SS. New data discussed here demonstrates that YopK also acts as a regulator of fidelity by preventing the export of middle Yops into host cells. How YopN and YopK coordinate these regulatory activities is unclear. Future endeavors will need to determine whether YopN and/or YopK are physically associated with the basal body and if so, with which proteins do they interact and are those interactions indeed required to regulate substrate specificity before and after cell contact? Likewise, YopK and YopE are both major regulators of translocation that function after they are injected into the host cell. YopE appears to exert its effect via manipulation of the actin cytoskeleton, while YopK associates with the translocation pore complex. How cytoskeletal changes influence the T3SS function, and whether these changes are channeled through the YopK-pore complex interaction is a mystery. Understanding the hierarchy and mechanisms of translocated regulatory proteins is an exciting new component of T3SS research.

### The two-step translocation model

A new model for Yop translocation has been suggested recently: the “two step” translocation model (Akopyan et al., 2011; Edgren et al., 2012). In this model, middle and late Yops can be secreted via the T3SS and subsequently associate with the bacterial outer membrane where they aggregate into complexes of translocators and effectors. These complexes would have to be released from bacteria, diffuse across the space between the bacterium and host cell, and then interact with membranes of target cells. Translocators YopB and YopD would then mediate transfer of the effector Yops into the host cell. In support of this model, Yops have been known to autoaggregate to the bacterial surface when triggered after secretion *in vitro* (Darveau et al., 1980; Straley and Brubaker, 1981; Bolin et al., 1982; Yother and Goguen, 1985). In fact, the binding of Yops to the surface of the bacterium before the T3SS was discovered led researchers to believe Yops were outer membrane proteins and hence their name, *Yersinia* outer-membrane proteins. It has also been shown that host cells can take up YopH-Bla coated on the surface of a Δ*yopH Y. pseudotuberculosis* mutant (Akopyan et al., 2011). Interestingly, the presence of the T3SS was necessary for this translocation to occur despite the fact that YopH-Bla was not expressed in the bacteria. This new “two step” model presents an interesting conundrum to the *Yersinia* field and to the research presented herein. Several questions remain unanswered. Do both methods of Yop delivery occur, and if so, which one is predominant *in vivo*? If the needle is not connected to the host as a continuous channel, then how is cell contact sensed in order to provide the critical signal to release the YopN regulatory blockade on late Yop secretion? If the injectisome is not involved in direct translocation of effectors into host cells, how are *Y. pestis* effectors YopK and YopE transmitting signals back to the bacteria? Do complexes of effector-Yops and translocator-Yops form pores that are subject to regulation by YopK, YopE, and YopT? It is difficult to envision a scenario in which a two-step delivery method would be compatible with the regulatory roles that YopK, YopE, and YopT seem to have within host cells. Data presented herein strongly implicate these bacterial effectors in generating a feedback signal that originates within host cells, which in turn implies that there is a continuous channel whose ends are able to communicate and exact precise control over the timing, specificity, and amplitude of Yop delivery. Of course such a channel remains hypothetical, and future work needs to place an emphasis on developing tools to allow visualization of actively translocating injectisomes docked onto host cells, as well as a more detailed view of the pore complex architecture. Such technological advances are crucial for providing insight into these key aspects of injectisome assembly and function.

### Conflict of interest statement

The authors declare that the research was conducted in the absence of any commercial or financial relationships that could be construed as a potential conflict of interest.
